# Decoding Critical Targets and Signaling Pathways in EBV-Mediated Diseases Using Large Language Models

**DOI:** 10.3390/v16111660

**Published:** 2024-10-24

**Authors:** Jingwen Yu, Yaohao Wang, Haidong Wang, Zhi Wei, Yonggang Pei

**Affiliations:** 1 School of Public Health and Emergency Management, Southern University of Science and Technology, Shenzhen 518055, China; 12233046@mail.sustech.edu.cn (J.Y.); 12231386@mail.sustech.edu.cn (Y.W.); wanghd@sustech.edu.cn (H.W.); 2 Department of Computer Science, New Jersey Institute of Technology, Newark, NJ 07102, USA; zhiwei@njit.edu

**Keywords:** EBV, signaling pathways, EBV-mediated diseases, LLM

## Abstract

Epstein–Barr virus (EBV), a member of the gamma herpesvirus, is the first identified human oncovirus and is associated with various malignancies. Understanding the intricate interactions between EBV antigens and cellular pathways is crucial to unraveling the molecular mechanisms in EBV-mediated diseases. However, fully elucidating EBV–host interactions and the associated pathogenesis remains a significant challenge. In this study, we employed large language models (LLMs) to screen 36,105 EBV-relevant scientific publications and summarize the current literature landscape on various EBV-associated diseases like Burkitt lymphoma (BL), diffuse large B-cell lymphoma (DLBCL), nasopharyngeal carcinoma (NPC), and so on. LLM-generated data indicate that the most-studied EBV-associated pathways are enriched in immune response, apoptosis, cell growth, and replication. The analyses of protein–protein interactions (PPIs) reveal three principal EBV-related protein clusters: TP53-centered apoptotic factors, EBV-associated transcription factors, and immune response elements. Utilizing our dataset and public databases, we demonstrated that BLLF3-targeted TLR2-associated factors are effective diagnostic markers for DLBCL. Next, we confirmed the co-expression of LMP1-targeted calcium pathway factors in BL. Finally, we demonstrated the correlation and co-expression of LMP1-induced *PARP1*, *HIF1A*, *HK2*, and key glycolysis-related factors, further suggesting that LMP1 actively regulates the glycolysis pathway. Therefore, our study presents a comprehensive functional encyclopedia of the interactions between EBV antigens and host signaling pathways across various EBV-associated diseases, providing valuable insights for the development of therapeutic strategies.

## 1. Introduction

Epstein–Barr virus (EBV) was discovered in 1964 and has infected over 90% of the world’s population [1]. The oncovirus contributes to various human diseases, such as Burkitt lymphoma (BL), Hodgkin lymphoma (HL), diffuse large-B cell lymphoma (DLBCL), nasopharyngeal carcinoma (NPC), gastric cancer (GC), post-transplant lymphoproliferative disease (PTLD), multiple sclerosis (MS), and so on. In the 60 years since its discovery, the intricate roles of EBV in regulating interconnected host cellular pathways have provided valuable insights into the mechanisms underlying EBV-related disease progression. EBV infection in human cells triggers a series of changes in cellular functions, including cell cycle transition, cell apoptosis, DNA mismatch repair, and multiple cellular signaling pathways. Such modulations are mediated by various EBV antigens, promoting viral latency and oncogenesis. For instance, EBV-encoded Latent Membrane Protein 1 (LMP1) mimics CD40 signaling to activate both the canonical and noncanonical NF-κB pathways [2,3,4,5]. EBV nuclear antigen 1 (EBNA1) inhibits the canonical NF-κB pathway by inhibiting the phosphorylation of IKKα/β [6]. Although the NF-κB pathway serves as a key factor in EBV latent infection and oncogenesis, the intricate and intertwined regulatory network of EBV antigens and NF-κB factors presents a significant challenge for the development of anti-EBV therapeutic strategies in EBV-related diseases. Therefore, it is imperative to comprehensively examine the landscape of current studies on EBV antigens and their interactions with cellular signaling pathways under certain EBV-related diseases to further explore EBV-mediated pathogenesis.

Traditional literature analysis techniques, including systematic reviews and meta-analyses, often fall short of capturing the full breadth of EBV literature. However, the rapid development in machine learning offers promising tools for analyzing critical factors in EBV-mediated pathogenesis from vast amounts of literature. A recent study characterized the biomedical literature into a 2D map [7]. The embedding contained 21 million papers, indicating the unlimited potential of LLM to study disease-specific metadata. Another work introduced a knowledge graph of the m6A regulatory pathway utilizing GPT-4 to elucidate m6A’s regulatory mechanisms in cancer phenotypes across various cancers [8]. In diseases with different EBV latency types, the expression of EBV antigens and their regulation of host gene expression vary under distinct conditions. Therefore, LLM bears the potential to identify and summarize the current functional impacts of various EBV antigens on host signaling pathways, which will provide easier access to literature references and enhance our current understanding of virus–host interactions in different diseases.

Our study aims to utilize LLMs to analyze decades of research on how EBV antigens regulate cellular factors and signaling pathways. We present a comprehensive framework of EBV antigens, their interacting host targets, and the associated pathways, constructing an extensive network that delineates the virus–host interaction in EBV-mediated diseases. Collectively, this study offers an overview of current EBV–host interactions and provides valuable insights for the development of disease-specific anti-EBV therapeutic strategies in the future.

## 2. Materials and Methods

### 2.1. Data Sources

The initial collections of available abstracts for this study were obtained from the PubMed database (https://pubmed.ncbi.nlm.nih.gov/) accessed on 28 February 2024. The search was performed using EDirect with the keyword combination “EBV OR HHV-4 OR human herpesvirus 4 OR Epstein-Barr virus”, resulting in a dataset of 36,105 EBV-related studies that includes only original articles and reviews published in English. Additionally, the RNA-seq dataset employed to validate differentially expressed genes induced by LMP1 overexpression was obtained from the Gene Expression Omnibus (GEO) database under accession number GSE247495.

### 2.2. ChatGPT-4 Prompt Engineering

The accuracy of the GPT-generated summary greatly depends on the concise prompt input with clear instructions, which can enhance the precision of the generated response. To assist GPT in recognizing EBV antigens, we provided ChatGPT with the lists of EBV antigens and EBV-related diseases through system messages prior to user dialogues (Table S1). The prompt for the antigen–target pathway summary is as follows: “If the abstract concludes that EBV-encoded proteins affect another protein or pathway, show me only the EBV protein, affected protein and pathway. If not, return ‘Error’ and nothing else. Use a temperature of 0. The abstract: XXX”. The prompt for the EBV-related disease mapping was “Return the specific EBV-related disease that the abstract attached in the end investigates. If unknown, return ‘Error’ and nothing else. The abstract: XXX”.

To optimize costs and efficiency, we initially utilized the free version GPT-3.5 Turbo via the OpenAI API using the prompts above. This would filter out articles unrelated to our theme, including reviews that do not contain conclusive relations between the EBV antigen and host signaling pathways in the abstracts. The remaining articles were then processed using GPT-4, which has a better performance for context summary. Finally, we removed error outputs and employed synonym substitution to standardize the naming of GPT-generated elements. For instance, “EBV nuclear antigen 1”, “EBNA1”, and “EBNA-1” are all annotated as “EBNA1”; “EB2”, “Mta”, “SM”, and “BS-MLF1” are all annotated as “BMLF1” (Table S2).

### 2.3. Evaluation of GPT-4 Generated Data

To evaluate the accuracy of the GPT-generated associations, we randomly selected a total of 115 entries (5% of the total) for manual evaluation (Table S3). The evaluation focused on the EBV antigens, the host factors that are associated with these antigens, and the GPT-generated pathways mentioned in the corresponding articles. The criteria for an “accurate result” are as follows: (1) the article clearly describes the interaction between the EBV antigen and its corresponding host factors; (2) the article identifies the biological changes influenced by this interaction and their relevance to the GPT-generated pathways. This screening does not distinguish between positive, negative, or structural regulations, and only serves to document interactions.

### 2.4. Annotation from GPT-Summarized Pathways to the KEGG Database

The GPT-generated pathways are highly variable and dependent on the corresponding abstracts. To establish a unique standard for statistical presentations, we annotated the GPT-generated pathways to the formal KEGG classification because of its simplicity and accessibility (Table S4). This mapping was performed based on our customized R script and empirical manual substitution.

### 2.5. Gene Enrichment Analysis

The annotation of GPT-generated host protein is performed using the R package STRINGdb (2.12.1). Annotated IDs are submitted to UniProt for symbol conversion (https://www.uniprot.org/, accessed on 20 April 2024). The generated list of genes of interest (GOIs) was subjected to gene enrichment analysis using three primary tools: the KEGG database [9], Reactome (https://reactome.org/, accessed on 25 April 2024), and STRINGdb (https://cn.string-db.org/, accessed on 20 April 2024). PPI networks are generated using the get_clusters function in the R package STRINGdb, and only relations with a confidence greater than 0.9 are included. We have provided original files of the PPI clusters with relational tables that can be viewed in Cytoscape (3.9.1) (Supplementary PPI 1–3).

### 2.6. Omics Analysis

Principal component analysis (PCA) and cohort analyses are performed using UCSC Xena [10] (https://xena.ucsc.edu/, accessed on 11 September 2024) and GEPIA2 (http://gepia2.cancer-pku.cn/, accessed on 11 September 2024) [11]. The dataset comprised TCGA Diffuse Large B-Cell Lymphoma, GTEx Spleen, and GTEx Blood. Differential gene expression analysis for the RNA-seq data is conducted using the R package DESeq2 (1.40.2). Genes with an adjusted *p*-value (p adj) of less than 0.05 and a log2 fold change greater than log2(2) were considered significantly differentially expressed.

### 2.7. Visualization

The figures are created using BioRender (https://biorender.com, accessed on 20 April 2024) or customized scripts with R packages such as wordcloud2 (0.2.1), circlize (0.4.16), and pheatmap (1.0.12). The PPI network is visualized in Cytoscape and colored according to protein family types. Other visualizations are made by a combination of R packages, including ggplot2 (3.5.0), ggalluvial (0.12.5), and enrichplot (1.20.3).

## 3. Results

### 3.1. LLMs-Assisted Data Mining Reveals the Key Insights in EBV Research

**Figure 1:**
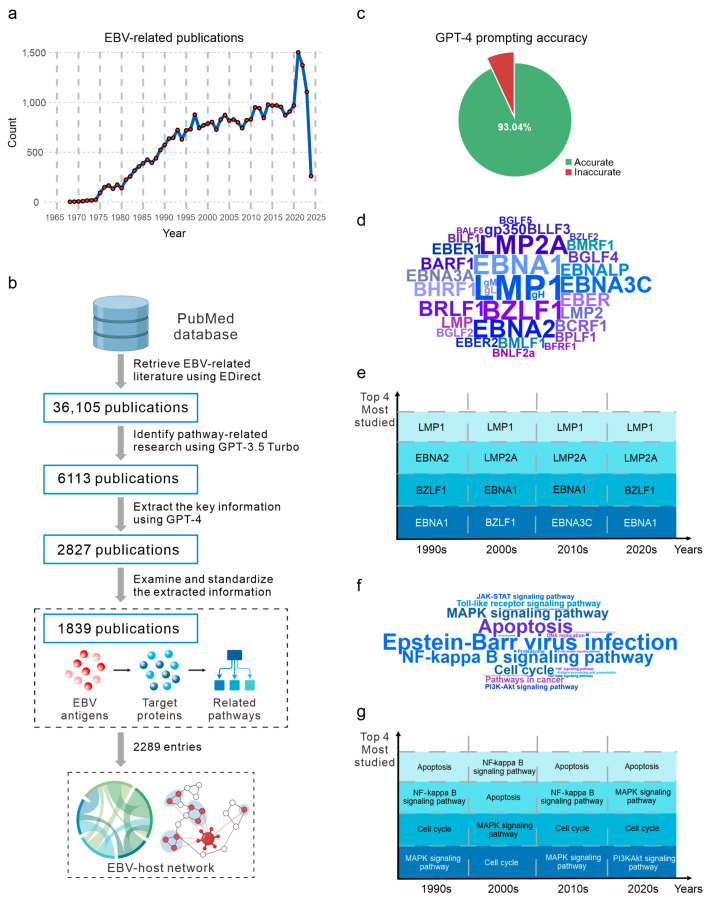
LLM-assisted data mining reveals the key insights in EBV research. (**a**) The trend of EBV-related publications in different years. (**b**) The pipeline in this study is illustrated as a flowgraph. (**c**) GPT-generated output is evaluated by examining 115 random entries. (**d**) The word cloud shows the relative frequency (log2 transformed) of research related to specific EBV antigens (LMP1, EBNA1, etc.). (**e**) Statistical analysis highlights the research focus on various EBV antigens over time, showcasing only the four most studied antigens for each decade. (**f**) The word cloud illustrates that the most numerous studies are centered on distinct signaling pathways mediated by NF-κB, MAPK, PI3K/Akt, and other factors. (**g**) Statistical analysis highlights the research focus on various EBV-related pathways over time, showcasing only the four most studied pathways for each decade.

To create a literature pool for information extraction, our initial dataset comprised 36,105 EBV-related research studies available in the PubMed database as of the start of this project (28 February 2024). These publications span a temporal range of 57 years, from 1968 to 2024 (Figure 1a). Overall, there has been a steady increase in the number of research articles related to EBV over these years, with an average of approximately 1000 publications annually.

Next, to further characterize the current EBV studies, we used ChatGPT-4 to summarize the available articles into a table, which includes PubMed ID, EBV antigens, target proteins, and the related signaling pathways. The returned dataset contained information from 2827 publications related to EBV antigen–pathway interactions and was subsequently subjected to manual data filtering (Figure 1b). The final dataset includes 2289 documented entries of EBV antigens and their associated pathways, derived from 1839 publications (Figure 1b, Table S5). The generation of inaccurate summaries of abstracts can be misleading. To validate the accuracy of the information extracted using ChatGPT, we manually compared a random subset of 115 entries (~5%) of the GPT-4 generated summaries with the corresponding original abstracts. The results indicate that our prompts achieved an average accuracy of 93.04% (Figure 1c, Table S3), demonstrating that our GPT-4-based pipeline can effectively obtain key information from the abstracts.

The analysis of EBV antigens and their impacts on cellular pathways reveals that LMP1 is the most frequently studied antigen, followed by EBNA1, BZLF1, LMP2A, and EBNA2, which are primarily latent or lytic proteins (Figure 1d). Throughout the 2010s, the significance of EBNA3C has increased, making it the fourth most studied antigen (Figure 1e). The most studied pathways focus on the mechanisms underlying EBV infection (Figure 1f). Pathways involving EBV-mediated cell apoptosis are the next most thoroughly studied areas. Abundant studies concentrate on the oncogenic mechanism of EBV infection, mainly on how EBV antigens regulate cell cycle and replication. The most extensively studied mechanisms include the NF-κB signaling pathway, MAPK signaling pathway, TLR signaling pathway, PI3K/Akt signaling pathway, TNF signaling pathway, and TGF-β signaling pathway (Figure 1f). On the temporal scale, research on cell apoptosis, the NF-κB signaling pathway, the cell cycle, and the MAPK signaling pathway have consistently remained among the four topics of greatest focus for researchers (Figure 1g), suggesting their critical roles in EBV-mediated diseases.

### 3.2. Pathway Mapping Reveals Distinct Signatures of EBV Antigens

**Figure 2:**
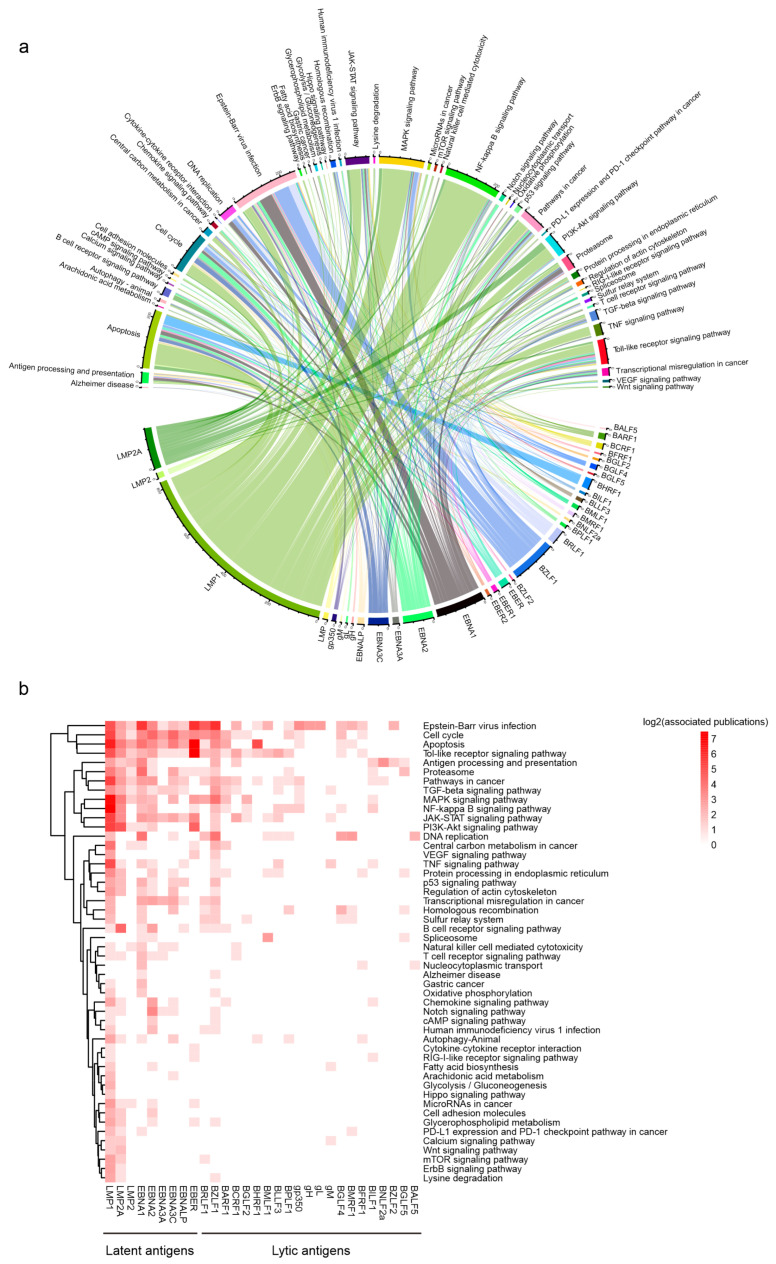
Pathway mapping reveals the distinct signatures of EBV antigens. (**a**) The chord diagram shows the linkage between the top 33 EBV antigens and their corresponding pathways. (**b**) Heatmap view of the linkage between EBV latent or lytic antigens and the associated pathways.

To clarify the critical pathways that EBV antigens regulate in EBV-related diseases, we annotated the GPT-generated pathways to the KEGG pathway entries (Table S4). The chord diagram denotes the frequency of the top 33 most studied EBV antigens and correlated pathways, indicating an intricate and interconnected network of cellular signaling pathways induced by EBV infection (Figure 2a). To clearly illustrate the concentrated interactions between EBV antigens and their related pathways, we present a chord diagram as a heatmap (Figure 2b). Then the pathways are clustered using the maximum linkage clustering method, and EBV antigens are arranged based on their roles in EBV latent or lytic infection. We observed that EBV latent membrane proteins, LMP1 and LMP2A, exhibit similar interaction patterns across different host cellular pathways, although the research on LMP1 has been more thorough, as previously discussed (Figure 1d). The resemblance between these two EBV antigens lies in the shared ability to reprogram the host immune response through the MAPK, PI3K/Akt, or TNF pathways, as well as their oncogenic functions in regulating the p53 or PD1/PD-L1 checkpoint pathway. Despite the significant scarcity of LMP2A-related studies compared to LMP1, the results collectively indicate that LMP2A contributes more to the BCR signaling pathway and the Wnt pathway than LMP1 (Figure 2b).

EBV nuclear antigens (EBNAs) contribute to the establishment and maintenance of viral latency, especially EBNA1, which is present in all EBV latent programs except for latency 0. EBNA1 directly binds to the host genome and is essential for the replication and maintenance of EBV episome [1]. It also serves as a transcription factor regulating multiple gene expression. Our analysis shows that EBNA1 differs from other EBNAs in terms of regulating DNA replication and oxidative phosphorylation (Figure 2b). EBNA2 plays a crucial role in the initial stage of the EBV-mediated B-cell transformation. Interestingly, EBNA2 has been confirmed to bind to super-enhancer (SE) sites that are critical for the maintenance of EBV latency [12,13]. Our findings highlight the distinctive roles of EBNA2 in cAMP signaling pathways, fatty acid biosynthesis, and cell adhesion lipid molecules, indicating its potential to facilitate B-cell transformation and proliferation through lipid metabolism (Figure 2b). Similar to EBNA1, the transcription factors EBNA3A and EBNA3C are shown to interfere with the host genome and promote immune evasion, cell cycle control, and inhibition of apoptosis. EBNALP binds to EBNA2 and functions as a coactivator to promote the transformation of B-cells during EBV infection [1]. Regarding the immediate-early lytic proteins, both BZLF1 and BRLF1 are well-studied and essential for EBV reactivation. Studies on BZLF1 seem more diverse on multiple cellular oncogenic pathways.

Other EBV antigens have received relatively less attention than the previously mentioned latent and lytic antigens (Table S6). EBV-encoded glycoproteins play important roles in facilitating entry into host cells during infection. Glycoprotein L (gL) cooperates with gH and gB to form the infection complex, mediating EBV-cell fusion and entry [1].

### 3.3. Gene Enrichment Uncovers Defining Characteristics of EBV-Induced Cellular Modulations

**Figure 3:**
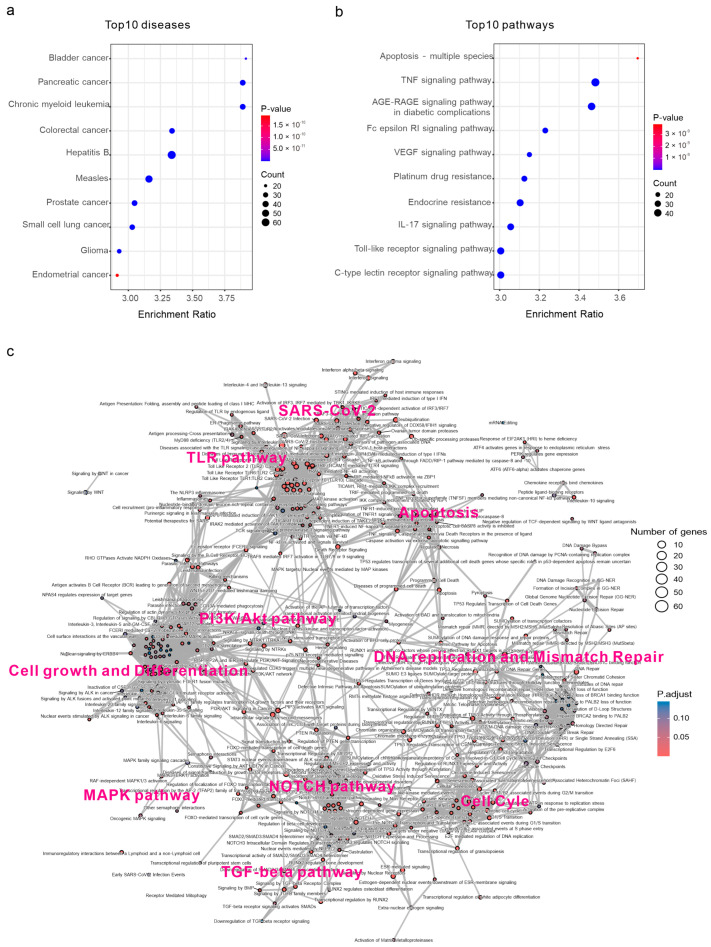
Gene enrichment and pathway analysis uncover defining characteristics of EBV-induced cellular modulations. (**a**,**b**) KEGG enrichment shows the top 10 EBV-related diseases or pathogens (**a**) and signaling pathways (**b**). (**c**) Reactome enrichment maps the landscape of EBV-targeted pathways and their correlations based on the shared host target proteins.

To map the landscape of the EBV-regulated host signaling network, we utilized GPT-generated host target proteins to perform the bioinformatic enrichment, and the KEGG enrichment shows the top 10 EBV-related diseases and pathways (Figure 3a,b). Furthermore, the pathway enrichment reveals the core EBV-related cellular signaling pathways, such as apoptosis, cell growth, cell cycle, and immune response (Figure 3c).

Disease-wise, KEGG enrichment revealed that EBV infection may be associated with traits typical of a wide range of diseases, including bladder cancer, pancreatic cancer, colorectal cancer, small cell lung cancer, and others (Figure 3a). The results suggest a possible correlation between these malignant diseases and EBV infection. However, it is important to note that the KEGG database does not include EBV-related diseases like Burkitt lymphoma. Consequently, the diseases highlighted are primarily autoimmune-related diseases and various forms of cancer (Figure 3a). The pathways enriched solely from target proteins and GPT-generated pathways exhibit notable similarities, with extensive studies on pathways such as apoptosis, the VEGF signaling pathway, the TNF signaling pathway, the Toll-like receptor signaling pathway, and the IL-17 signaling pathway (Figure 3b). This shows the coherence between GPT-generated pathways and the original studies. The last three pathways highlight the importance of studying how EBV antigens modulate innate immunity. The IL-17 signaling pathway triggers the downstream NF-κB signaling pathway and is known to be a key pathogenic factor of autoimmune diseases [14]. However, the relationship between the IL-17 pathway and EBV infection remains unexplored. The high expression of IL-17 in multiple sclerosis patients could present a novel area of EBV research and provide druggable candidates for certain autoimmune diseases [15].

To provide a more comprehensive overview of the functions of EBV-targeted cellular proteins, we used the Reactome database for annotation and identified 375 biological processes (*p* < 0.05) related to EBV infection (Figure 3c). This network shows that EBV-targeted host proteins predominantly form clusters in approximately 10 functional pathway groups. The largest clusters include innate immunity pathways, represented by the TNF signaling pathway, Toll-like receptor signaling cascades, and NF-κB signaling pathways. These are followed by cell growth and differentiation pathways, exemplified by the PI3K/AKT signaling pathways and related factors such as interleukins. Subsequently, DNA replication and mismatch repair, along with cell cycle-related pathways, are prominently featured, particularly those regulated by TP53. Moreover, the NOTCH signaling pathway and its regulation of the TGF-β signaling pathways mediated by the SMAD proteins are also critical for cell proliferation, differentiation, transitions, and apoptosis in our model of cellular signaling crosstalk [16].

Intriguingly, we have identified some active mediators bridging the interconnected cellular signaling pathways (Figure 3c). These active members include deubiquitination and sumoylation, which regulate protein modification and proteostasis; TP53-mediated cellular senescence on DNA replication and cell cycle; PTEN and downstream AKT signaling activation mediated by PIP3; BCR signaling for multiple pathways spanning the immune response to cell proliferation; and Fc epsilon receptor (FCERI) signaling for the same function. Targets related to SARS-CoV-2 infection are enriched alongside the immune signaling pathways, suggesting a shared immune response elicited by EBV and SARS-CoV-2. This indicates the potential for developing broad-spectrum antivirals for both viruses by targeting shared cellular factors. Additionally, more discrete, smaller clusters were identified, including the Wnt signaling pathway, mRNA editing, and the unfolded protein response (UPR), which is essential for maintaining cellular homeostasis and promoting cell survival during protein misfolding and ER stress.

### 3.4. Analysis of Host Protein–Protein Interactions Identifies Key Mediators in EBV-Associated Diseases

**Figure 4:**
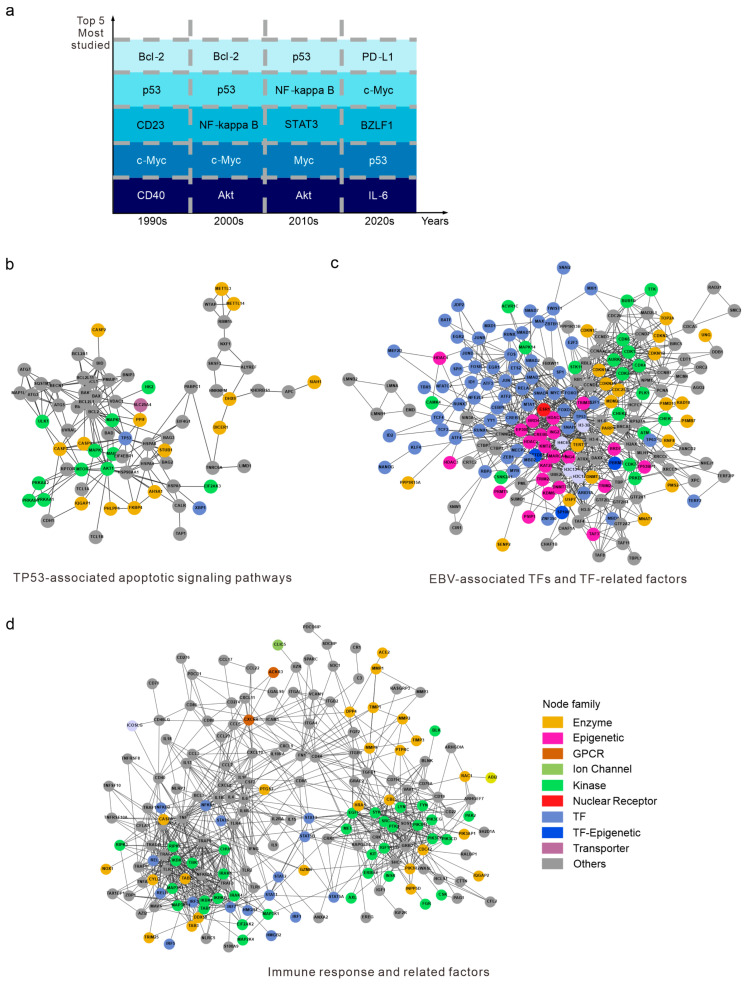
Analysis of host protein-protein interactions identifies key mediators in EBV-associated diseases. (**a**) Statistical analysis reveals the focus of study on EBV-targeted host proteins in each decade. TF, transcription factor; GPCR, G Protein-Coupled Receptor. (**b**–**d**) The top three PPI networks describe the primary functional clusters of EBV-targeted host proteins.

To further characterize the critical cellular factors involved in EBV-associated diseases, we explored the EBV-targeted host proteins independent of pathway enrichment, which can provide insights into EBV-modulated key proteins. The bibliographic analysis of publications over time reveals trends in EBV-related host proteins (Figure 4a). p53 and c-Myc are the two most studied proteins across all periods. Genomic profiling has identified frequent *TP53* [17,18,19] and *MYC* [20,21] mutations or rearrangements in EBV-positive malignancies. CD23 and CD40 are two critical factors for EBV infection on the B-cell membrane. Earlier studies identified that EBNA2 specifically induces CD23 expression vital for B-cell transformation [22], and they verified the role of LMP1 in viral latency maintenance via mimicry of the CD40 signaling pathways [23]. As previously discussed in Figure 2, multiple EBV antigens, including BHRF1 and LMP1, are associated with Bcl-2-related apoptosis in lymphomas and immortalized B-cells [19,24,25].

To better characterize the pathways and networks of EBV-related host factors described above, we annotated all the target proteins using the STRING database and performed protein–protein interaction analysis. By examining the statistical data regarding the research focus on EBV-targeted host proteins over the decades, we identified three major EBV-associated clusters, which demonstrate EBV-targeted proteins and the available interactive factors (Figure 4b–d, Supplementary PPI 1–3). The first PPI cluster centers around TP53-associated genes, apoptotic pathways, the mTOR pathway, and MAPK pathway cascades along with gene silencing mediated by DICER1 and m6A methylation (Figure 4b, Supplementary PPI 1). This cluster highlights the roles of EBV in suppressing the host apoptotic pathways and related signal transduction pathways to promote viral latency and oncogenesis. Clinical trials and genomic profiling studies have identified the downregulation and mutation of *TP53* in multiple EBV-associated cancers [26,27,28,29]. EBV-encoded microRNAs (EBERs) are shown to maintain Burkitt lymphomas by inhibiting caspase 3-mediated apoptosis and facilitating the transformation of naïve B-cells [30]. Such inhibition usually works in harmony with other factors like Bcl-2. In addition to triggering classic apoptotic pathways, EBV also evades the host antiviral systems by decreasing the host Dicer protein through EBNA1-mediated microRNA degradation [31]. Recent studies have also uncovered the role of circular RNAs in promoting the progression of EBV-associated gastric carcinoma through the transactivation of METTL3 [32]. In this pathway, caspases have been found to inhibit the m6A RNA modification process, thereby promoting EBV replication [33]. Additionally, EBV infection is known to induce TCL1 gene expression in Burkitt lymphoma, which also serves as an activator of the Akt pathway [34].

The second cluster highlights EBV’s roles in transcriptional regulation and epigenetic modification, particularly through transcriptional factors (TFs) (Figure 4c, Supplementary PPI 2). MYC is a key TF associated with multiple cancers including EBV-associated lymphomas [35]. Moreover, MYC abundance represses EBV reactivation and controls the lytic switch in Burkitt lymphoma [36]. 3D genome mapping of EBV-positive lymphoblastoid cell lines (LCLs) revealed distinctive EBV super-enhancer (SE) targets like MCL1, IRF4, and EBF [37]. Bioinformatic analysis identified that H3K27ac SEs overlap with STAT5 and NFAT targets, indicating their roles in promoting EBV latency [13]. On a larger scale, a broad screening of TFs integrated with over 700 high-throughput sequencing datasets provided an atlas of EBV-related transcriptomic and epigenetic host–virus regulatory interactions [38]. These EBV-specific TFs, such as SPI1, EBF1, and PAX5, collectively contribute to EBV-induced latency and oncogenesis [13].

The last cluster primarily comprises factors related to the innate immune response, including cellular receptors, cytokines, and chemokines that regulate immune cell activation, the inflammatory response, and the tumor microenvironment (TME) (Figure 4d, Supplementary PPI 3). EBV orchestrates immune evasion by cellular receptors like CD40, CD80, IL4, IL6, IL10, and their ligands [39,40]. For example, LMP1 mimics CD40 to activate downstream NF-κB pathways to induce B-cell proliferation [23]. After infection, EBV hijacks host signaling transduction factors like STATs [41,42] and IRFs [43] to promote cell survival and proliferation. LMP1 and LMP2A then activate the JAK/STAT pathway to facilitate cell growth factors and anti-apoptotic signaling. Meanwhile, EBV and other herpesviruses induce the PI3K/Akt pathway to facilitate viral infection, latency, and reactivation [44]. This cluster is also rich in enzymes, multiple cellular growth factors (CGFs), and extracellular matrix (ECM) proteins, which represent the invasiveness of EBV-infected cells. Early research discovered that viral LMP1 and BZLF1 proteins upregulate the expression and activity of matrix metalloproteinase 1 (MMP1), and thereby confer the invasive properties of the cells [45]. Besides common growth factors, we also identified Angiotensin-Converting Enzyme 2 (ACE2) in this cluster. A study indicates that the lytic replication of EBV induces ACE2 expression in human epithelial cells, which enhances the entry of SARS-CoV-2 [46].

### 3.5. Exploring the Critical Relationships in EBV-Mediated Diseases

**Figure 5:**
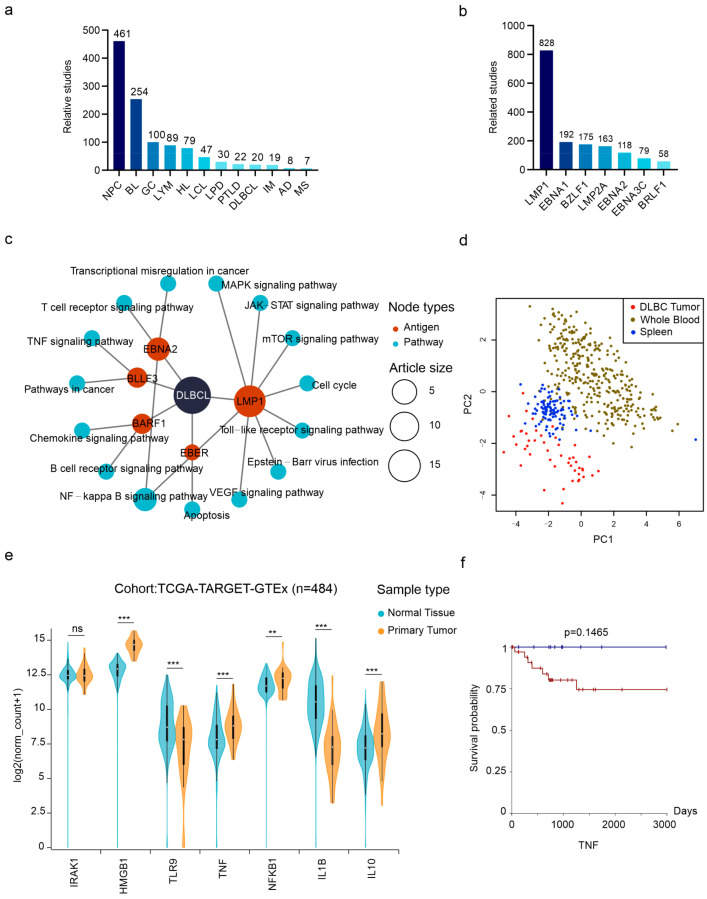
Exploring the critical relationships in EBV-mediated DLBCLs. (**a**) The bar plot shows the number of total articles on the top 12 most studied EBV-related diseases. NPC, nasopharyngeal carcinoma; BL, Burkitt lymphoma; GC, gastric carcinoma; LYM, lymphoma; HL, Hodgkin’s lymphoma; LCL, lymphoblastoid cell line; LPD, lymphoproliferative disease; PTLD, post-transplant lymphoproliferative disease; DLBCL, diffuse large B-cell lymphoma; IM, infectious mononucleosis; AD, Alzheimer’s disease; MS, multiple sclerosis. (**b**) The bar plot shows the total articles of the top 7 most studied EBV antigens. (**c**) The circular hub graph elucidates the DLBCL-related antigen–pathway relations. (**d**) PCA analysis highlights distinct DLBCL tumor samples using TLR2-related marker gene expressions. (**e**) The TLR2-related marker gene expressions between normal tissue and tumor tissue. (**, *p* < 0.01; ***, *p* < 0.001; ns, not significant) (**f**) The survival plot shows high TNF expression leads to less survival probability. The red line indicates samples with normalized TNF counts ≥ 7.907 (n = 36), and blue indicates less (n = 11).

**Figure 6:**
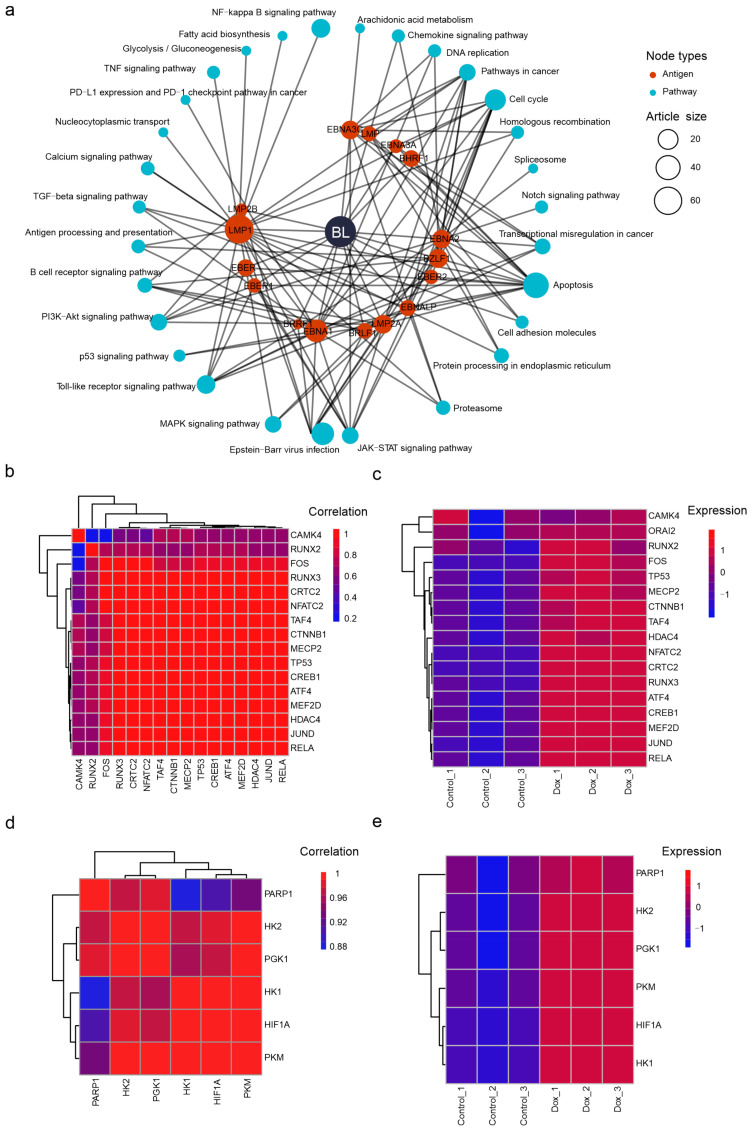
Exploring the critical relationships in EBV-mediated BL. (**a**) The circular hub graph elucidates the BL-related antigen-pathway relations. (**b**,**c**) The CAMK4-related factors are co-expressed by LMP1 overexpression. (**d**,**e**) The glycolysis-related factors are co-expressed by LMP1 overexpression.

Due to the complex background of EBV pathogenesis, we next seek to elucidate the effects of EBV antigens on host signaling pathways in the context of multiple EBV-associated malignancies (Figure 5a,b). Additionally, we demonstrate how our dataset and generated PPI networks can assist EBV–host interaction studies using multiple omics analyses. We plot the connections identified for EBV antigens and host pathways onto circular connection hubs for DLBCL, BL, NPC, GC, and PTLD (Figure 5c, Figure 6a, and Figure S1).

Approximately 9% of DLBCLs are EBV-positive with type II latency [1]. In DLBCL-centered hubs, we noticed the special linkage of EBV-encoded dUTPase BLLF3 and the TNF signaling pathways (Figure 5c). This indicates that BLLF3 induces DLBCL through the TNF pathways. dUTPase is crucial for maintaining the balance of intracellular nucleotides, particularly during DNA replication [47]. Our dataset highlights a study exploring BLLF3 and its interaction with host factor TLR2 [48]. Our PPI networks show several known key factors closely interacting with TLR2 in the background of EBV infection, such as IRAK1, HMGB1, TLR9, TNF, NFKB1, IL1B, and IL10. Next, we conducted a pan-cancer analysis to examine the utility of these proteins in distinguishing between DLBCL and normal samples (Figure 5d). The results indicated a clear difference between the tumor and samples from normal tissues, suggesting their potential as marker genes for DLBCL diagnosis. Due to the absence of corresponding gene expression data for normal tissue in TCGA for DLBCL, we used whole blood and spleen as normal samples for comparison. Taken apart, we noticed the high expression of HMGB1, TNF, NFKB1, and IL10 in DLBCL samples, all of which function in the TNF pathway (Figure 5e). At last, we examined the correlation between TNF expression and the survival probability of DLBCL patients. The results show that the survival possibility of patients with high TNF expression dropped to 75% in approximately four years while samples with low expression survived 100% (Figure 5f). These findings suggest the potential of exploring BLLF3-mediated activation of the TNF pathway in the carcinogenesis of DLBCL.

LMP1 is the most studied EBV antigens in our literature pool (Figure 5b). In BL, the literature indicates that LMP1 activates the calcium signaling pathway by overexpressing the host factor CaM kinase-Gr (CAMK4) [49] (Figure 6a, Table S5). Our PPI network reveals that HDAC4 and CREB1 directly interact with CAMK4, and they are also associated with other cellular factors, such as MEF2D, RUNX3, RUNX2, NFATC2, JUND, FOS, RELA, TP53, TAF4, MECP2, CTNNB1, ATF4, and CRTC2 (Figure 4c, Supplementary PPI 2). Although a dox-induced overexpression of LMP1 in the Akata cells did not result in a significant increase in CAMK4, its interacting proteins listed above exhibited a co-expression pattern and consistent upregulation (Figure 6b,c). This suggests that the calcium signaling pathway may contribute to the oncogenesis of BL in an LMP1-dependent manner.

Finally, we identified studies that investigate similar pathways involving the same antigens across cell lines from various EBV-related malignancies to enhance the utility. For instance, these studies on BL and NPC both demonstrated that LMP1 is associated with glycolysis (Figure 6a and Figure S1a). Overexpressing LMP1 in BL cell lines suggests that LMP1 activates PARP1 to induce glycolysis and promote oncogenesis in a HIF-1α-dependent manner [50]. A study on NPC cell lines demonstrated that LMP1 activates HK2 expression to elevate glycolysis and promote cell proliferation by blocking apoptosis [51]. These studies on cell lines with different EBV infection statuses demonstrate a more comprehensive role of LMP1 in regulating glycolysis. Next, we explore the transcriptomic correlation for PARP1, HIF1A, HK2, and key factors in glycolysis, such as PGK1, PKM, and HK1 (Figure 6d,e). These results indicate a high correlation and collective activation of these genes, which suggests a more complete mechanism in which LMP1 regulates the reprogramming of EBV-mediated glycolysis.

To conclude, we have illustrated the application of our dataset using three instances. First, DLBCL-specific studies show the importance of TLR2-related factors targeted by BLLF3 in the diagnosis of DLBCL. Second, the protein interactions depicted in PPI networks are confirmed to have similar transcriptional signatures, demonstrating the effectiveness of our PPI network. Finally, the studies conducted on different cell lines have elucidated a more comprehensive understanding of how LMP1 reprograms glycolytic metabolism. Collectively, these instances demonstrate the usability and practicality of our dataset as a functional encyclopedia, detailing the associations between EBV antigens, host factors, and signaling pathways.

## 4. Discussion

Our study presents a novel in silico approach using large language models (LLMs) to summarize the available studies associated with EBV antigens and cellular signaling pathways in EBV-related diseases. Next, we used bioinformatic tools to annotate the critical antigen–pathway relations and characterize the critical EBV-targeted proteins within functional clusters in PPI networks. Finally, we combined the online pan-cancer database and RNA-seq data to validate the interesting findings in our screen.

However, several limitations and concerns still need to be addressed in the future. While our initial data pool contained 36,105 EBV-related publications, our strict prompts and data filtering reduced this to 2289 entries, encompassing 1839 unique publications (~5.1%) for downstream analyses. If we loosen the criteria, we can obtain more entries for deeper analyses, but the accuracy may decrease. Therefore, it will be expected to simultaneously analyze more studies with advanced methods while ensuring accuracy. Secondly, our literature study was based solely on conclusions drawn from abstracts of the available publications by LLMs, which has its intrinsic limitation in the study of network biology. Although we used the frequency of topics as a proxy for their importance in the EBV antigen-pathway mapping, the sheer quantity of studies may not fully represent actual biological significance, so further experiments are necessary to validate these findings. Furthermore, EBV latency types are critical in understanding EBV gene expression and virus–host interactions, but such information is largely unattainable in abstracts. Future research using more powerful tools can be applied to extract the main text for related information.

Additionally, the PPI relations are from the STRING protein database with a confidence greater than 0.9. However, these relations do not elaborate on structural affinity or conformational changes in these proteins due to the complexity of protein interactions and the difficulty in analyzing the vast literature pool. To address this caveat, future efforts to employ LLMs for EBV research may focus on supervised multi-modal machine learning algorithms, which can be used to explore the expression changes or structural interactions between EBV antigens and EBV-induced cellular changes to enhance utility and usability.

This study represents a pioneering effort in applying LLMs to EBV-related research, highlighting the potential of LLMs in advancing our understanding of EBV–host interactions. Unlike broad-scale context generation, our study clarifies the relationships between EBV antigens and various cellular pathways based on the manual annotation of GPT-generated summaries, presenting relations between EBV antigens and host proteins in various EBV-related diseases for future research. Beyond EBV, the big data era and advancements in various LLMs underscore the urgent need for advanced, digitized platforms in all areas of viral research. Such tools are pivotal for accelerating research progress, advancing basic virology, and fostering the development of novel antivirals.

## 5. Conclusions

This study, for the first time, presents a mapping of EBV antigens, host factors, and cellular pathways from available EBV-related studies using LLMs. We first mapped the relations between EBV antigens and related pathways independent of disease background to show the collective studies for each antigen. Next, the pathway enrichment network demonstrates that current studies on EBV-related pathways center around immune response, cell growth, cell cycle, apoptosis, and DNA replication. PPI analysis reveals three principal EBV-related functional protein clusters: TP53-centered apoptotic factors, EBV-associated transcription factors, and immune response elements. Finally, we present the disease–antigen–pathway relations for DLBCL, BL, NPC, PTLD, and GC. Combined with omics databases, our screen identified TLR2-related factors as potential diagnostic markers for DLBCL tumors. We then validated the effectiveness of our PPI network using calcium factors and LMP1-overexpressing BL cells. Finally, based on shared studies indicating the role of LMP1 in regulating glycolysis in BL and NPC, we confirmed the co-expression of PARP1, HIF1A, HK2, and key glycolysis factors induced by LMP1.

## Data Availability

Data are contained within the article and Supplementary Materials.

## Supplementary Materials

The following supporting information can be downloaded at: https://www.mdpi.com/article/10.3390/v16111660/s1, Figure S1: Determining the key relationships in EBV-mediated NPC, PTLD, and GC. (a–c) The circular hub graph elucidates the NPC-related, PTLD-related, and GC-related antigen–pathway relations. NPC, nasopharyngeal carcinoma; PTLD, Posttransplant lymphoproliferative disorders; GC, gastric cancer; Table S1: The EBV antigens and EBV-associated diseases sent to LLMs in system messages; Table S2: The formal names and aliases used in GPT-generated term substitution; Table S3: Validation for GPT-generated results; Table S4: The correspondence between GPT-4 generated pathways and manually annotated pathway to KEGG entries; Table S5: The final dataset includes 2289 entries of EBV antigens and their associated pathways; Table S6: Ranking of the most studied EBV antigens (with >10 related articles); Supplementary PPI 1: PPI cluster for TP53-associated apoptotic signaling pathways; Supplementary PPI 2: PPI cluster for the EBV-associated TFs and TF-related factors; Supplementary PPI 3: PPI cluster for the immune response and related factors.

## References

[1] Damania B., Kenney S.C., Raab-Traub N. (2022). Epstein-Barr Virus: Biology and Clinical Disease. Cell.

[2] Hatzivassiliou E., Miller W.E., Raab-Traub N., Kieff E., Mosialos G. (1998). A Fusion of the EBV Latent Membrane Protein-1 (LMP1) Transmembrane Domains to the CD40 Cytoplasmic Domain Is Similar to LMP1 in Constitutive Activation of Epidermal Growth Factor Receptor Expression, Nuclear Factor-Kappa B, and Stress-Activated Protein Kinase. J. Immunol..

[3] Kaykas A., Sugden B. (2000). The Amino-Terminus and Membrane-Spanning Domains of LMP-1 Inhibit Cell Proliferation. Oncogene.

[4] Gewurz B.E., Towfic F., Mar J.C., Shinners N.P., Takasaki K., Zhao B., Cahir-McFarland E.D., Quackenbush J., Xavier R.J., Kieff E. (2012). Genome-Wide siRNA Screen for Mediators of NF-κB Activation. Proc. Natl. Acad. Sci. USA.

[5] Luftig M., Prinarakis E., Yasui T., Tsichritzis T., Cahir-McFarland E., Inoue J.-I., Nakano H., Mak T.W., Yeh W.-C., Li X. (2003). Epstein–Barr Virus Latent Membrane Protein 1 Activation of NF-κB through IRAK1 and TRAF6. Proc. Natl. Acad. Sci. USA.

[6] Valentine R., Dawson C.W., Hu C., Shah K.M., Owen T.J., Date K.L., Maia S.P., Shao J., Arrand J.R., Young L.S. (2010). Epstein-Barr Virus-Encoded EBNA1 Inhibits the Canonical NF-κB Pathway in Carcinoma Cells by Inhibiting IKK Phosphorylation. Mol. Cancer.

[7] González-Márquez R., Schmidt L., Schmidt B.M., Berens P., Kobak D. (2024). The Landscape of Biomedical Research. Patterns.

[8] Wu X., Zeng Y., Das A., Jo S., Zhang T., Patel P., Zhang J., Gao S.-J., Pratt D., Chiu Y.-C. (2024). reguloGPT: Harnessing GPT for Knowledge Graph Construction of Molecular Regulatory Pathways. bioRxiv.

[9] Kanehisa M., Goto S. (2000). KEGG: Kyoto Encyclopedia of Genes and Genomes. Nucleic Acids Res..

[10] Goldman M.J., Craft B., Hastie M., Repečka K., McDade F., Kamath A., Banerjee A., Luo Y., Rogers D., Brooks A.N. (2020). Visualizing and Interpreting Cancer Genomics Data via the Xena Platform. Nat. Biotechnol..

[11] Tang Z., Kang B., Li C., Chen T., Zhang Z. (2019). GEPIA2: An Enhanced Web Server for Large-Scale Expression Profiling and Interactive Analysis. Nucleic Acids Res..

[12] Bal E., Kumar R., Hadigol M., Holmes A.B., Hilton L.K., Loh J.W., Dreval K., Wong J.C.H., Vlasevska S., Corinaldesi C. (2022). Super-Enhancer Hypermutation Alters Oncogene Expression in B Cell Lymphoma. Nature.

[13] Zhou H., Schmidt S.C.S., Jiang S., Willox B., Bernhardt K., Liang J., Johannsen E.C., Kharchenko P., Gewurz B.E., Kieff E. (2015). Epstein-Barr Virus Oncoprotein Super-Enhancers Control B Cell Growth. Cell Host Microbe.

[14] Mills K.H.G. (2023). IL-17 and IL-17-Producing Cells in Protection versus Pathology. Nat. Rev. Immunol..

[15] Luchtman D.W., Ellwardt E., Larochelle C., Zipp F. (2014). IL-17 and Related Cytokines Involved in the Pathology and Immunotherapy of Multiple Sclerosis: Current and Future Developments. Cytokine Growth Factor. Rev..

[16] Luo K. (2017). Signaling Cross Talk between TGF-β/Smad and Other Signaling Pathways. Cold Spring Harb. Perspect. Biol..

[17] Menter T., Juskevicius D., Alikian M., Steiger J., Dirnhofer S., Tzankov A., Naresh K.N. (2017). Mutational Landscape of B-Cell Post-Transplant Lymphoproliferative Disorders. Br. J. Haematol..

[18] Bruce J.P., To K.-F., Lui V.W.Y., Chung G.T.Y., Chan Y.-Y., Tsang C.M., Yip K.Y., Ma B.B.Y., Woo J.K.S., Hui E.P. (2021). Whole-Genome Profiling of Nasopharyngeal Carcinoma Reveals Viral-Host Co-Operation in Inflammatory NF-κB Activation and Immune Escape. Nat. Commun..

[19] Frontzek F., Staiger A.M., Wullenkord R., Grau M., Zapukhlyak M., Kurz K.S., Horn H., Erdmann T., Fend F., Richter J. (2023). Molecular Profiling of EBV Associated Diffuse Large B-Cell Lymphoma. Leukemia.

[20] Panea R.I., Love C.L., Shingleton J.R., Reddy A., Bailey J.A., Moormann A.M., Otieno J.A., Ong’echa J.M., Oduor C.I., Schroeder K.M.S. (2019). The Whole-Genome Landscape of Burkitt Lymphoma Subtypes. Blood.

[21] Garcia-Reyero J., Martinez Magunacelaya N., Gonzalez de Villambrosia S., Loghavi S., Gomez Mediavilla A., Tonda R., Beltran S., Gut M., Pereña Gonzalez A., d’Ámore E. (2021). Genetic Lesions in MYC and STAT3 Drive Oncogenic Transcription Factor Overexpression in Plasmablastic Lymphoma. Haematologica.

[22] Wang F., Gregory C.D., Rowe M., Rickinson A.B., Wang D., Birkenbach M., Kikutani H., Kishimoto T., Kieff E. (1987). Epstein-Barr Virus Nuclear Antigen 2 Specifically Induces Expression of the B-Cell Activation Antigen CD23. Proc. Natl. Acad. Sci. USA.

[23] Uchida J., Yasui T., Takaoka-Shichijo Y., Muraoka M., Kulwichit W., Raab-Traub N., Kikutani H. (1999). Mimicry of CD40 Signals by Epstein-Barr Virus LMP1 in B Lymphocyte Responses. Science.

[24] Oudejans J.J., van den Brule A.J., Jiwa N.M., de Bruin P.C., Ossenkoppele G.J., van der Valk P., Walboomers J.M., Meijer C.J. (1995). BHRF1, the Epstein-Barr Virus (EBV) Homologue of the BCL-2 Protooncogene, Is Transcribed in EBV-Associated B-Cell Lymphomas and in Reactive Lymphocytes. Blood.

[25] Kenney J.L., Guinness M.E., Curiel T., Lacy J. (1998). Antisense to the Epstein-Barr Virus (EBV)-Encoded Latent Membrane Protein 1 (LMP-1) Suppresses LMP-1 and Bcl-2 Expression and Promotes Apoptosis in EBV-Immortalized B Cells. Blood.

[26] Ribeiro J., Malta M., Galaghar A., Silva F., Afonso L.P., Medeiros R., Sousa H. (2017). P53 Deregulation in Epstein-Barr Virus-Associated Gastric Cancer. Cancer Lett..

[27] Sarkozy C., Hung S.S., Chavez E.A., Duns G., Takata K., Chong L.C., Aoki T., Jiang A., Miyata-Takata T., Telenius A. (2021). Mutational Landscape of Gray Zone Lymphoma. Blood.

[28] Lee J.E., Choi Y.Y., An J.Y., Kim K.T., Shin S.-J., Cheong J.-H. (2022). Clinicopathologic and Genomic Characteristics of Mucinous Gastric Adenocarcinoma. Gastric Cancer.

[29] Lee H.S., Chang M.S., Yang H.-K., Lee B.L., Kim W.H. (2004). Epstein-Barr Virus-Positive Gastric Carcinoma Has a Distinct Protein Expression Profile in Comparison with Epstein-Barr Virus-Negative Carcinoma. Clin. Cancer Res..

[30] Vereide D.T., Seto E., Chiu Y.-F., Hayes M., Tagawa T., Grundhoff A., Hammerschmidt W., Sugden B. (2014). Epstein-Barr Virus Maintains Lymphomas via Its miRNAs. Oncogene.

[31] Mansouri S., Pan Q., Blencowe B.J., Claycomb J.M., Frappier L. (2014). Epstein-Barr Virus EBNA1 Protein Regulates Viral Latency through Effects on Let-7 microRNA and Dicer. J. Virol..

[32] Zhang J.-Y., Du Y., Gong L.-P., Shao Y.-T., Pan L.-J., Feng Z.-Y., Pan Y.-H., Huang J.-T., Wen J.-Y., Sun L.-P. (2022). Ebv-circRPMS1 Promotes the Progression of EBV-Associated Gastric Carcinoma via Sam68-Dependent Activation of METTL3. Cancer Lett..

[33] Zhang K., Zhang Y., Maharjan Y., Sugiokto F.G., Wan J., Li R. (2021). Caspases Switch off the m^6^A RNA Modification Pathway to Foster the Replication of a Ubiquitous Human Tumor Virus. mBio.

[34] Bell A., Rickinson A.B. (2003). Epstein-Barr Virus, the TCL-1 Oncogene and Burkitt’s Lymphoma. Trends Microbiol..

[35] Dhanasekaran R., Deutzmann A., Mahauad-Fernandez W.D., Hansen A.S., Gouw A.M., Felsher D.W. (2022). The MYC Oncogene—The Grand Orchestrator of Cancer Growth and Immune Evasion. Nat. Rev. Clin. Oncol..

[36] Guo R., Jiang C., Zhang Y., Govande A., Trudeau S.J., Chen F., Fry C.J., Puri R., Wolinsky E., Schineller M. (2020). MYC Controls the Epstein-Barr Virus Lytic Switch. Mol. Cell.

[37] Jiang S., Zhou H., Liang J., Gerdt C., Wang C., Ke L., Schmidt S.C.S., Narita Y., Ma Y., Wang S. (2017). The Epstein-Barr Virus Regulome in Lymphoblastoid Cells. Cell Host Microbe.

[38] Arvey A., Tempera I., Tsai K., Chen H.-S., Tikhmyanova N., Klichinsky M., Leslie C., Lieberman P.M. (2012). An Atlas of the Epstein-Barr Virus Transcriptome and Epigenome Reveals Host-Virus Regulatory Interactions. Cell Host Microbe.

[39] Park G.B., Kim Y.S., Lee H.-K., Cho D.-H., Kim D., Hur D.Y. (2013). CD80 (B7.1) and CD86 (B7.2) Induce EBV-Transformed B Cell Apoptosis through the Fas/FasL Pathway. Int. J. Oncol..

[40] Kis L.L., Takahara M., Nagy N., Klein G., Klein E. (2006). IL-10 Can Induce the Expression of EBV-Encoded Latent Membrane Protein-1 (LMP-1) in the Absence of EBNA-2 in B Lymphocytes and in Burkitt Lymphoma- and NK Lymphoma-Derived Cell Lines. Blood.

[41] Weber-Nordt R.M., Egen C., Wehinger J., Ludwig W., Gouilleux-Gruart V., Mertelsmann R., Finke J. (1996). Constitutive Activation of STAT Proteins in Primary Lymphoid and Myeloid Leukemia Cells and in Epstein-Barr Virus (EBV)-Related Lymphoma Cell Lines. Blood.

[42] Hsiao J.-R., Jin Y.-T., Tsai S.-T., Shiau A.-L., Wu C.-L., Su W.-C. (2003). Constitutive Activation of STAT3 and STAT5 Is Present in the Majority of Nasopharyngeal Carcinoma and Correlates with Better Prognosis. Br. J. Cancer.

[43] Wang L., Ren J., Li G., Moorman J.P., Yao Z.Q., Ning S. (2017). LMP1 Signaling Pathway Activates IRF4 in Latent EBV Infection and a Positive Circuit between PI3K and Src Is Required. Oncogene.

[44] Cohen J.I. (2018). Herpesviruses in the Activated Phosphatidylinositol-3-Kinase-δ Syndrome. Front. Immunol..

[45] Lu J., Chua H.-H., Chen S.-Y., Chen J.-Y., Tsai C.-H. (2003). Regulation of Matrix Metalloproteinase-1 by Epstein-Barr Virus Proteins. Cancer Res..

[46] Verma D., Church T.M., Swaminathan S. (2021). Epstein-Barr Virus Lytic Replication Induces ACE2 Expression and Enhances SARS-CoV-2 Pseudotyped Virus Entry in Epithelial Cells. J. Virol..

[47] Fleischmann J., Kremmer E., Greenspan J.S., Grässer F.A., Niedobitek G. (2002). Expression of Viral and Human dUTPase in Epstein-Barr Virus-associated Diseases. J. Med. Virol..

[48] Williams M., Ariza M.E. (2018). EBV Positive Diffuse Large B Cell Lymphoma and Chronic Lymphocytic Leukemia Patients Exhibit Increased Anti-dUTPase Antibodies. Cancers.

[49] Mosialos G., Hanissian S.H., Jawahar S., Vara L., Kieff E., Chatila T.A. (1994). A Ca2+/Calmodulin-Dependent Protein Kinase, CaM Kinase-Gr, Expressed after Transformation of Primary Human B Lymphocytes by Epstein-Barr Virus (EBV) Is Induced by the EBV Oncogene LMP1. J. Virol..

[50] Hulse M., Caruso L.B., Madzo J., Tan Y., Johnson S., Tempera I. (2018). Poly(ADP-Ribose) Polymerase 1 Is Necessary for Coactivating Hypoxia-Inducible Factor-1-Dependent Gene Expression by Epstein-Barr Virus Latent Membrane Protein 1. PLoS Pathog..

[51] Xiao L., Hu Z.-Y., Dong X., Tan Z., Li W., Tang M., Chen L., Yang L., Tao Y., Jiang Y. (2014). Targeting Epstein-Barr Virus Oncoprotein LMP1-Mediated Glycolysis Sensitizes Nasopharyngeal Carcinoma to Radiation Therapy. Oncogene.

