# Supplementary figures and images for: Decoding Critical Targets and Signaling Pathways in EBV-Mediated Diseases Using Large Language Models
Source: Viruses. 2024 Oct 24;16(11):1660. doi: 10.3390/v16111660 (PMC11598986; doi:10.3390/v16111660)

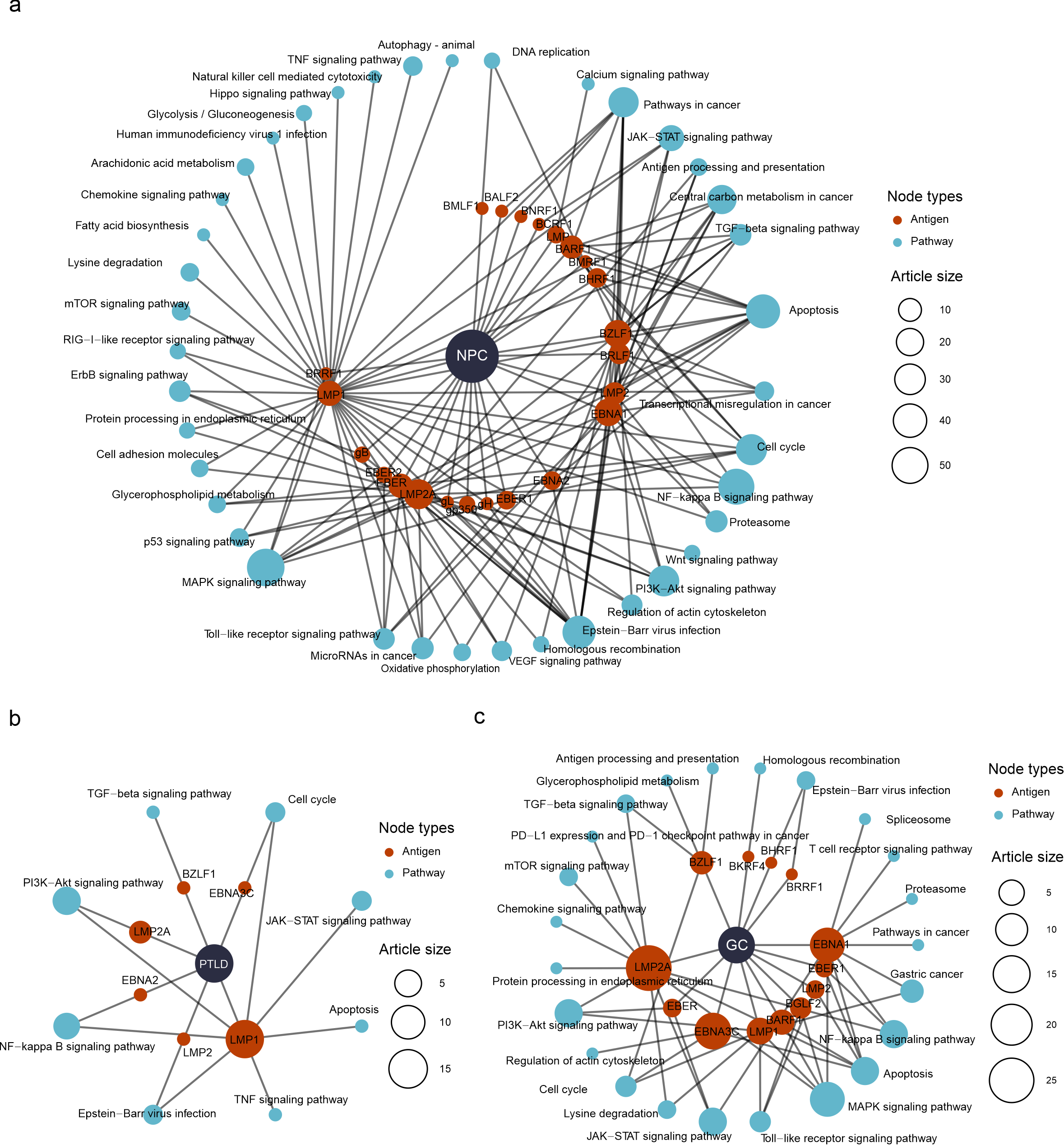

Supplement: Supplementary file 1 [file viruses-16-01660-s001.zip › Supplementaries/Figure S1.tif]
